# Lightwave nano-converging enhancement by an arrayed optical antenna based on metallic nano-cone-tips for CMOS imaging detection

**DOI:** 10.1038/s41598-022-20077-y

**Published:** 2022-09-21

**Authors:** Chai Hu, Taige Liu, Kewei Liu, Jiashuo Shi, Mao Ye, Xinyu Zhang

**Affiliations:** 1 National Key Laboratory of Science & Technology On Multispectral Information Processing, Huazhong University of Science & Technology, Wuhan, 430074 China; 2 School of Automation & Artificial Intelligence, Huazhong University of Science & Technology, Wuhan, 430074 China; 3 Innovation Institute, Huazhong University of Science & Technology, Wuhan, 430074 China

**Keywords:** Optics and photonics, Optical materials and structures, Nanoparticles

## Abstract

A kind of gold-coated glass nano-cone-tips (GGNCTs) is developed as an arrayed optical antenna for highly receiving and converging incident lightwaves. A local light field enhancement factor (LFEF) of ~ 2 × 10^4^ and maximum light absorption of ~ 98% can be achieved. The near-field lightwave measurements at the wavelength of 633 nm show that the surface net charges over a single GGNCT make a typical dipole oscillation and the energy transmits along the wave vector orientation, thus leading to a strong local light field enhancement. An effective detection method by near-field coupling an arrayed GGNCT and complementary metal–oxide–semiconductor (CMOS) sensor for highly efficient imaging detection is proposed. The lightwave detection at several wavelengths, including typical 473 nm, 532 nm, 671 nm, and 980 nm, shows a notable characteristic that a better capability of the net charge distribution adjusting and localized aggregating can be obtained at the absorption peak of the GGNCT developed and a stronger signal detection achieved. The research lays a foundation for further developing a light detector with an ideal optoelectronic sensitivity and broad spectral suitability, which is based on integrating GGNCTs as an arrayed optical antenna with common sensors.

## Introduction

As known, the surface plasmons (SPs) mainly originated from the interaction between surface free electrons and incident lightwaves, which can be efficiently excited over the surface or interface of some metallic or semiconductive materials. Under the condition of the SP frequency being the same as that of the incident lightwaves and also the momentum conservation satisfied, a steady SP resonance can be predicted^1–3^. So, a reasonable design of the functioned micro-nano-architectures over the surface of several common metallic or semiconductive structures can be performed for efficiently adjusting the regional SP energy and momentum distribution and even their surface transportation stimulated, which means a feasible approach to efficiently control or finely adjust the directional propagation and then the spatial resonant aggregation of the surface electromagnetic waves in a specific band^4–6^. The SP converging towards a metal nano-tip enables a highly localized and then remarkably enhanced light field in a nanoscale region^7–9^. However, the local light field enhancement factor (LFEF, |***E***|^2^/|***E***_0_|^2^) of the SP resonance is often limited due to intrinsic dissipative and radiative losses^10^. The plasmonic nanoparticle structures with a high-LFEF resonance and also suppressed loss would be highly beneficial to many applications that require a sharp resonant response and an intense enhancement of light-matter interaction. Generally, the SP nano-focusing not only relies on the interaction of the surface resonant waves excited and the patterned micro-nano-structures constructed, but also is defined as a transmission phenomenon^11–14^. Thus, the upright optical nano-antenna array is proposed compared to the common planar nano-antenna^15^. According to a featured fashion of the surface electron density waves, the SPs will propagate in a specific polarization orientation of incident lightwaves towards the apex of a single nano-tip. As the effective dimension of a metallic tip is smaller, the SPs can be more efficiently guided and then squeezed into a nanoscale space such as the apex, and thus presenting a very strong near-field lightwave resonant enhancement based on a highly tighter light field confinement of nano-tips^16–19^, which is generally determined by the sharpness of the nano-tip and also the Coulomb blockade from the apex as a special quantum point^20–24^.

Detectors with several typical performances such as high spatial resolution, high sensitivity, and very large array scale, are urgently desired in a large number of high-tech applications and manufacturing industry, for example, in both the civilian and defense fields, especially in modern medical imaging and artificial intelligence^25–27^. In recent years, the development of high-sensitivity photodetectors based on the localized light-field enhancement antenna has gradually become a hot research topic^28–30^. So far, several representative nanostructural photodetectors with some attractive characteristics, for instance, high photoconductive gain, controllable wavelength sensitivity, fast response, and highly efficient light-to-current conversion, have been realized and also demonstrated a remarkable optoelectronic characteristic of tunable light absorption and high carrier mobility^31^. This paper proposes a kind of gold-coated glass nano-cone-tips (GGNCTs), as an arrayed optical antenna for highly efficient receiving and converging incident lightwaves, so as to achieve a typical high LFEF of ~ 2 × 10^4^. The upright GGNCT arrays with different structural sizes are fabricated. As demonstrated experimentally, the surface free electrons of the GGNCTs can be effectively excited by the lasers with a central wavelength of 633 nm and then present a dipole of net charge oscillation along the direction of the incident ***E***-field. Generally, the apex structure means a substantial surface net charge aggregating and then light energy accumulating, which should be more efficient corresponding to the GGNCTs with a larger cone sharpness, and thus eventually realize a powerful ***E***-field enhancement effect. It should be noted that when the LFEF and absorption reach the maximum at the same time, a strong convergence effect will be produced at the top of the cone. Based on the property, a new detection method by coupling the optical antenna composed of a large number of nano-cone-tips with COMS sensors is proposed for achieving a very weak lightwave detection and opto-signal amplification. It highlights the continuous development of the highly efficient infrared photosensitive technique.

## Results and discussion

### Numerical simulation

**Figure 1:**
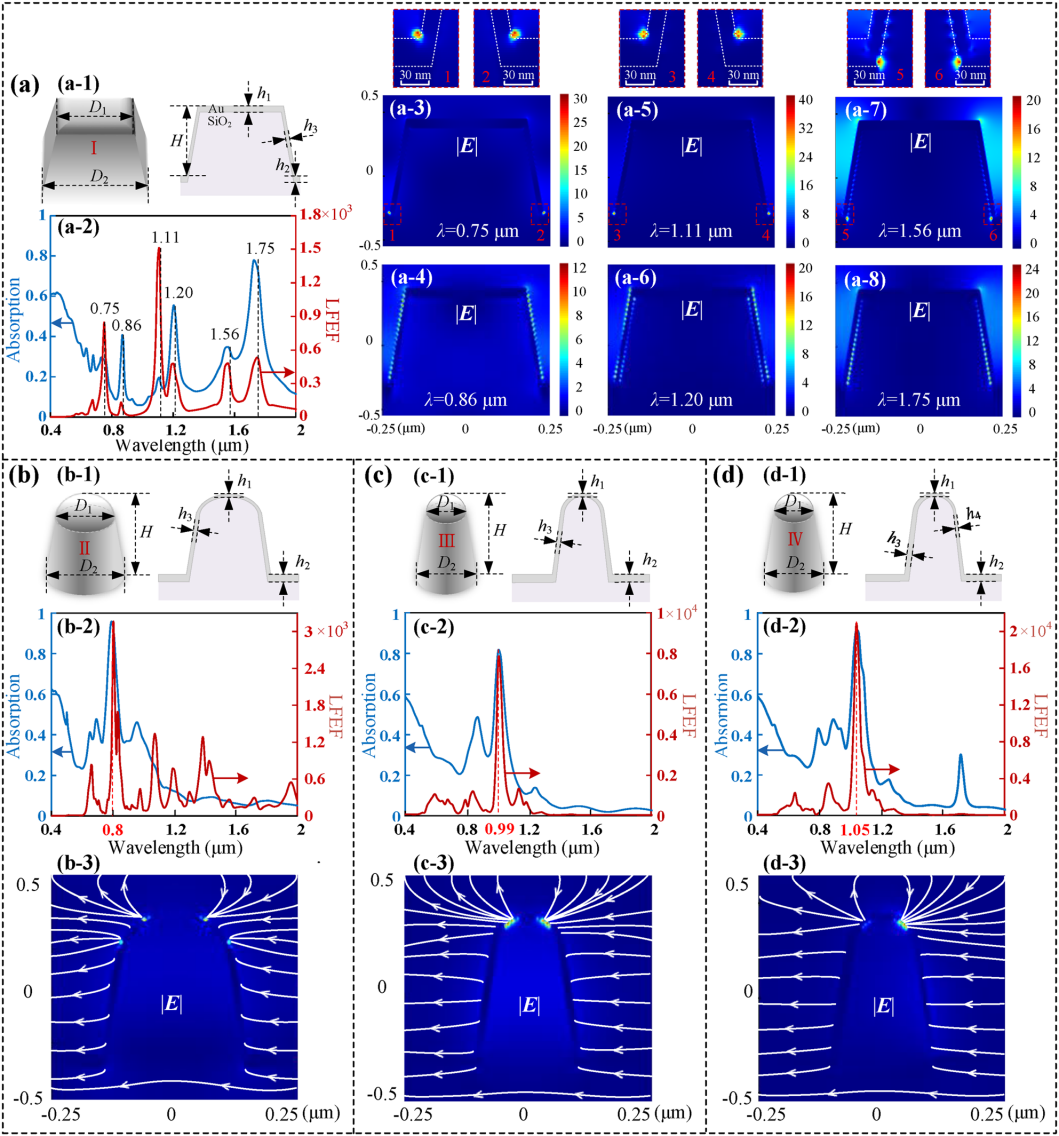
Simulations of the GGNCTs with the lightbeams in a wavelength range of 0.4–2 μm and illuminating normally from the bottom. (**a**) The main simulation results of GGNCT-I. (**a-1**) Two-layered SiO_2_ and metal films with critical parameters. The top and bottom side lengths are *D*_1_ = 375 nm and *D*_2_ = 460 nm, and the height *H* = 300 nm. The thicknesses of the gold film at the top and bottom are *h*_1_ = *h*_2_ = 30 nm and at the sidewalls *h*_3_ = 15 nm. (**a-2**) The LFEF and the light absorption curves. (**a-3**)–(**a-8**) The |***E|*** cross-sectional profiles of several light wavelengths are selected. The enlarged views of the strong near-field convergence spot demonstrated in (**a-3**), (**a-5**), and (**a-7**), are inserted above the corresponding figures. (**b**–**d**) Structural parameters and simulations for a single GGNCT with a large base area (**b**), and a small base area (**c**), and an asymmetrical metal coating (**d**). Structural parameters for (**b-1**): The top and bottom diameters are *D*_1_ = 215 nm and *D*_2_ = 300 nm, and the height *H* = 300 nm. The thicknesses of the gold film at the top and bottom are *h*_1_ = 10 nm and *h*_2_ = 30 nm, and at the sidewalls *h*_3_ = 15 nm; Structural parameters for (**c-1**): The top and bottom diameters are *D*_1_ = 145 nm and *D*_2_ = 225 nm, and the height *H* = 300 nm. The thicknesses of the gold film at the top and bottom are *h*_1_ = 10 nm and *h*_2_ = 30 nm, and at the sidewalls *h*_3_ = 15 nm; Structural parameters for (**d-1**): The top and bottom diameters are *D*_1_ = 145 nm and *D*_2_ = 225 nm, and the height *H* = 300 nm. The thicknesses of the gold film at the top and bottom are *h*_1_ = 10 nm and *h*_2_ = 30 nm, and at the left and right sidewalls *h*_3_ = 20 nm and *h*_4_ = 10 nm. (**b-2**)–(**d-2**) The LFEF and the light absorption curves, and (**b-3**)–(**d-3**) show the typical ***E***-field distributions at the resonance wavelengths. The instantaneous ***E***-field directions are represented by white streamlines.

**Figure 2:**
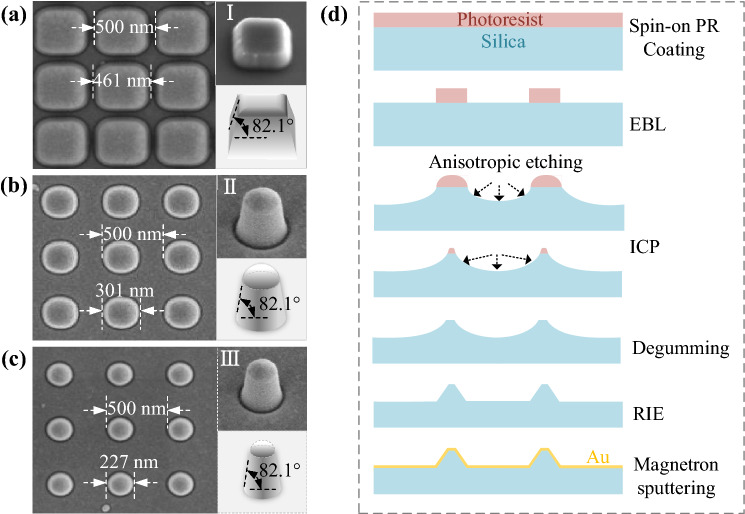
SEM micrographs of several GGNCT arrays with different morphology and the process flow. The main structural parameters (*S*_*T*_, *D*_1_, *θ*) of the GGNCTs are as follows: (**a**) (500, 461, 82.1°), (**b**) (500, 301, 82.1°), and (**c**) (500, 227, 82.1°). (**d**) The key fabrication processes including typical EBL, ICP, and RIE.

**Figure 3:**
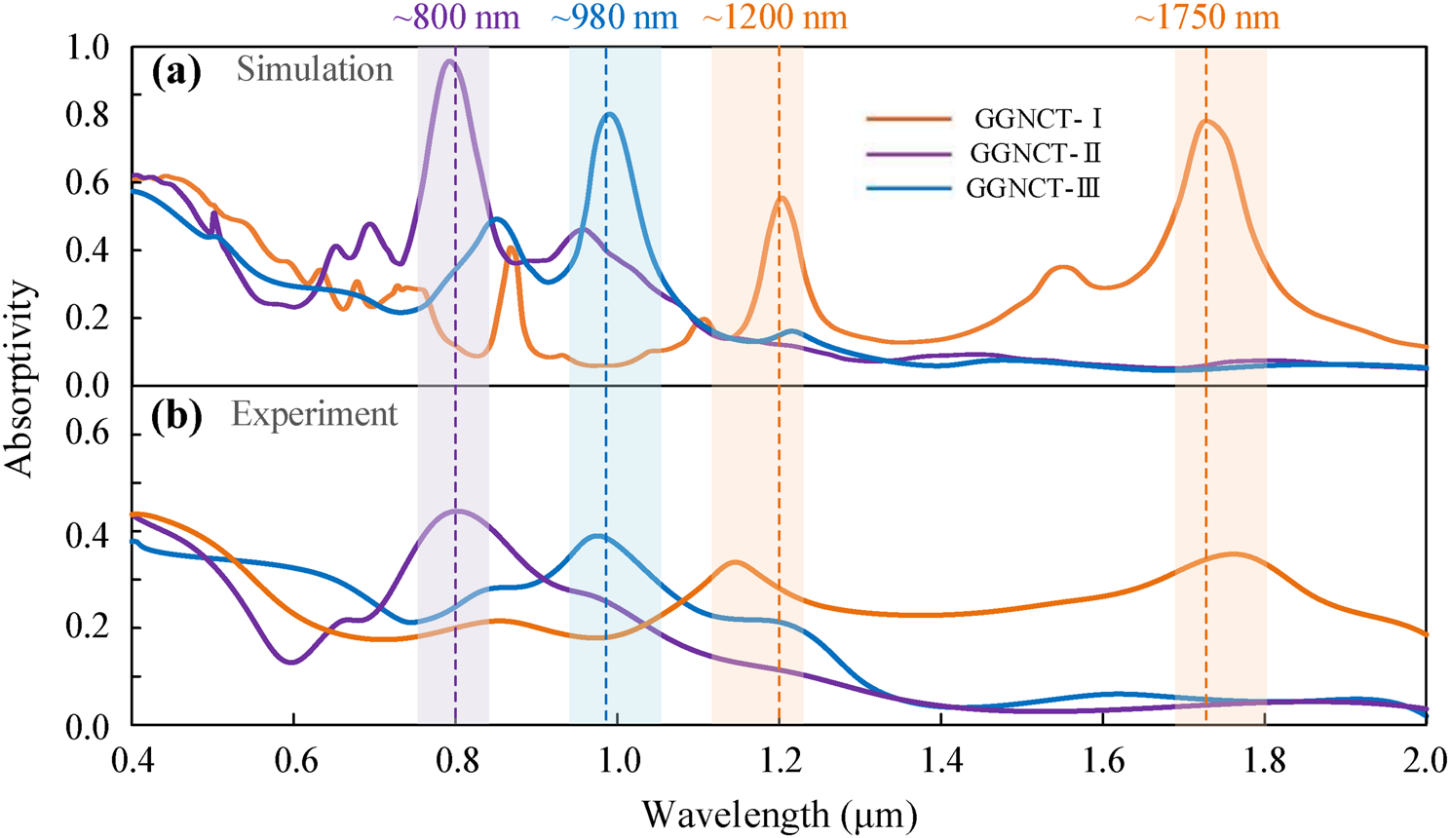
The simulated and measured absorption spectrums of the GGNCT-I and II and III in a wavelength range of 0.4–2 μm, which are colored by orange, purple, and blue, respectively. (**a**) The simulated absorption spectrums and (**b**) the measured absorption spectrums.

Several two-layered structures composed of glass wafer and gold film, including GGNCT-I, II, III, and IV, are modeled and simulated by FDTD Lumerical Solutions. The main parameters and the simulations are shown in Fig. 1. Considering a functional architecture with a finite structural size and suitable to periodic boundary conditions at the *x*- and *y*-axes so as to avoid complex edge effects, the perfectly matched layer (PML) boundary configuration is selected corresponding to the *z*-axis. And the simulation period is set as 500 nm and the plane lightwaves with a wavelength range from 0.4 to 2 μm are normally incident from the bottom. According to the memory size of the simulation files and the reliability of the structural model, the mesh division accuracy is 2 nm, 2 nm, and 2 nm in the *x*-, *y*-, and *z*-axes, respectively. The frequency-domain field and power monitors are used to simulate the reflectivity and transmissivity. Both the reflection (*R*) and transmission (*T*) components can be obtained by performing direct measurements, and thus the absorption part can be easily converted according to the relation of *A* = 1 − *R* − *T*. The frequency-domain field profile monitor is set for obtaining the featured electric field distribution of the nano-cone-tip. Figure 1a shows a typical cone-shaped GGNCT-I with main structural parameters. As shown in Fig. 1(a-1), the top and bottom side lengths are 375 nm and 460 nm, and the height is 300 nm. The thickness of the gold film at the top and bottom is 30 nm and at the sidewalls is 15 nm. Figure 1(a-2) shows the typical situations of the light absorption and the LFEF indicated by the blue and red curves, such as significant resonance peaks at six wavelengths including 0.75 μm, 0.86 μm, 1.11 μm, 1.20 μm, 1.56 μm, and 1.75 μm. The tip of the nanostructure can effectively converge the near-field light waves leading to large field enhancement. Thus, the tip with the strongest local light field energy enhancement factor LFEF in the whole nanostructure is the focus of the study. Therefore, the maximum LFEF at each wavelength, which is also the maximum value in the colorbar of the electric field distribution profiles, is chosen to plot the LFEF curve. The maximum absorption occurs at 1.75 μm and exceeds a high value of 0.8. But the maximum LFEF appears at 1.11 μm and reaches 1500. The ***E***-field distribution at each peak wavelength is given for exploring the resonance peak formation. As shown in Figs. 1(a-3)–(a-8), both absorption peak and LFEF peak can lead to a convergence effect. At the wavelengths of 0.75 μm and 1.11 μm, the LFEF is relatively large but the absorptivity does not show a remarkable increase, where the* E*-field localized at the bottom of GGNCT-I. At the wavelengths of 0.86 μm and 1.2 μm and 1.75 μm, the absorption is comparatively large but the LFEF has a lower peak, where the ***E***-field is distributed on the sidewalls of GGNCT-I. At the wavelength of 1.56 μm, it is not shown a relatively great enhancement of both the light absorption and the LFEF, where the ***E***-fields are arranged at both the bottom and the sidewalls.

By adjusting the structural parameters to obtain more desirable results about the ***E***-field strongly localized at the top of the GGNCT, so as to maximize the light absorption and the LFEF, a cone-shaped GGNCT-II is designed, as shown in Fig. 1b. The main structural parameters are demonstrated in Fig. 1(b-1). The top and the bottom diameter are 215 nm and 300 nm, and the height is 300 nm. The thickness of the gold film formed at the top and the bottom and the sidewalls are 10 nm and 30 nm and 15 nm. The simulations are shown in Fig. 1(b-2). As demonstrated, both the light absorption and the LFEF curves present the maximum resonance peaks (~ 0.95, ~ 3300) at the wavelength of 0.8 μm. Figure 1(b-3) shows the ***E***-field distribution at the resonance wavelengths. The instantaneous ***E***-field directions are represented by white streamlines. The ***E***-field profile shows the electric dipole distribution at the top of the cone in the direction of incident ***E***-field polarization.

The case of further shrinking the lateral dimension of the cone to result in a sharper cone-shaped GGNCT-III is shown in Fig. 1c. As shown in Fig. 1(c-1), the key parameters including the top and bottom diameter and the height are 145 nm and 225 nm and 300 nm. The thickness of the gold film at the top and the bottom and the sidewalls are 10 nm and 30 nm and 15 nm. As shown in Fig. 1(c-2), both the light absorption and the LFEF curves present the maximum resonance peak (~ 0.82, ~ 8000) at the wavelength of 0.99 μm, and a couple more robust electric dipole is formed at the GGNCT top, as shown in Fig. 1(c-3). Furthermore, the case through adjusting the surface gold film of the GGNCT-III with an asymmetric coating, so as to result in the film thicknesses of the left sidewall being 20 nm and the right being 10 nm, is shown in Fig. 1d. The key parameters of GGNCT-IV are shown in Fig. 1(d-1). The asymmetric mode is achieved by slightly moving the inner glass nano-cone-tip towards the right by 5 nm based on the symmetric mode during simulations. Similarly, both the light absorption and the LFEF curves will obtain the maximum resonance peak (~ 0.98, ~ 2 × 10^4^) at the wavelength of 1.05 μm, as shown in Fig. 1(d-2). The ***E***-field is mainly localized at the top of the thinner sidewall leading to a significantly robust bright spot, as shown in Fig. 1(d-3).

In summary, it can be seen that when LFEF and absorption reach the maximum at the same time, a strong convergence effect will be produced at the top of the cone, and the peak wavelength will gradually redshift with the reduction of the lateral size of GGNCT. Moreover, the larger cone sharpness can make the local field enhancement effect more significant. We can predict that the local field enhancement phenomenon should be caused by the transmission of SP on the surface of the nano-cone-tip from the bottom to the top along the wave vector orientation. And the asymmetric coating method has a better effect than the symmetric coating. It is known that at the metal-air interface, the net free-electron oscillations are directly affected by the incident electric field component. The SPs excited on both sides of a symmetric nanostructure are in anti-phase according to the polarization orientation of the incident electric field component^32^. For the symmetric coating method, the SPs destructive interference occurs at the top of a single nano-cone-tip. The asymmetric coating will eliminate the SPs destructive interference, and thus lead to a strong near-field nano-focusing.

### Absorption spectrum experiment

Considering the feasibility of the current process, the symmetric coating method is chosen to produce the nano-cone-tip-tips. According to the target design, the anisotropic plasmonic metasurface is fabricated and then the featured optical character is also characterized. The GGNCTs-I, -II, and -III are fabricated mainly based on a critical anisotropic etching on a ~ 500 μm thick glass wafer. Then a gold film is evaporated over the patterned surface of the glass wafer. The surface morphology of the obtained GGNCTs is characterized by a traditional scanning electron microscope (SEM), as shown in Fig. 2. The top-view of the GGNCTs is exhibited in Fig. 2a–c, and the 3D stereogram of each GGNCT is observed by tilting the samples at 30°, 30°, and 5°, which are inserted in the upper right corner. The corresponding structural models from the same viewpoint are given in the lower right corner for ease of viewing. The acquired GGNCTs are then characterized by featured parameters including the spatial period *S*_*T*_ of 500 nm and the cone bottom diameters *D*_1_ of ~ 227 nm, ~ 301 nm, and ~ 461 nm. And the GGNCTs with the same height of ~ 300 nm and a similar sidewall with a slope *θ* of ~ 82.1°.

The main technological processes are shown in Fig. 2d. Firstly, a layer of negative photoresist (Ma-N2403) is coated over the glass substrate with a sprayed aluminum film as a conductive layer. Then, the standard electron beam lithography (JBX 6300FS, JEOL) is utilized to shape a fine resist. Furthermore, the inductively coupled plasma etching (Oxford Plasma Pro System 100 ICP 380) is used to perform anisotropic etching in a mixed atmosphere composed of C_4_F_8_ and O_2_. After degumming, the reactive ion etching (ME-3A) is conducted to shape a relatively sharp sidewall. Finally, the magnetron sputtering (Kurt J. Lesker LAB 18) is utilized to deposit a 30 nm thick gold film over the patterned surface of the glass substrate with a 5 nm thick titanium as an adhesive. Since the side wall of the nano-cone is relatively steep, and the sputtered titanium on the side wall is thinner and cannot form a film, so most areas are still basically in contact with the silicon substrate. Therefore, the influence of titanium was not considered in the simulation.

The simulated absorption spectrums demonstrated in Figs. 1(a-2)–(c-2) are picked out together for comparative analysis, as shown in Fig. 3a, where the curves colored by orange and purple and blue represent the absorption spectrum of the GGNCTs-I, -II, and -III, respectively. The dispersive spectrometer (Model iHR550 of HORIBA), has the following key parameters: the focal length of 550 mm, the resolution of 0.06 nm, the grating size of 76 mm × 76 mm, and a spectral range of 0.4 to 2.0 μm. The reflection (*R*) and transmission (*T*) components can be acquired by direct measurements, and the absorption part easily be converted according to the relation of *A* = 1-*R*-*T*. The experimental absorption curves are shown in Fig. 3b. As demonstrated, the simulated and measured absorptivity curves follow a similar trend, where the GGNCT-I exhibits absorption peaks near ~ 1200 nm and ~ 1750 nm, and the GGNCT-II an absorption peak near ~ 800 nm, and the GGNCT-III an absorption peak near ~ 980 nm.

### Near-field simulation and measurement

**Figure 4:**
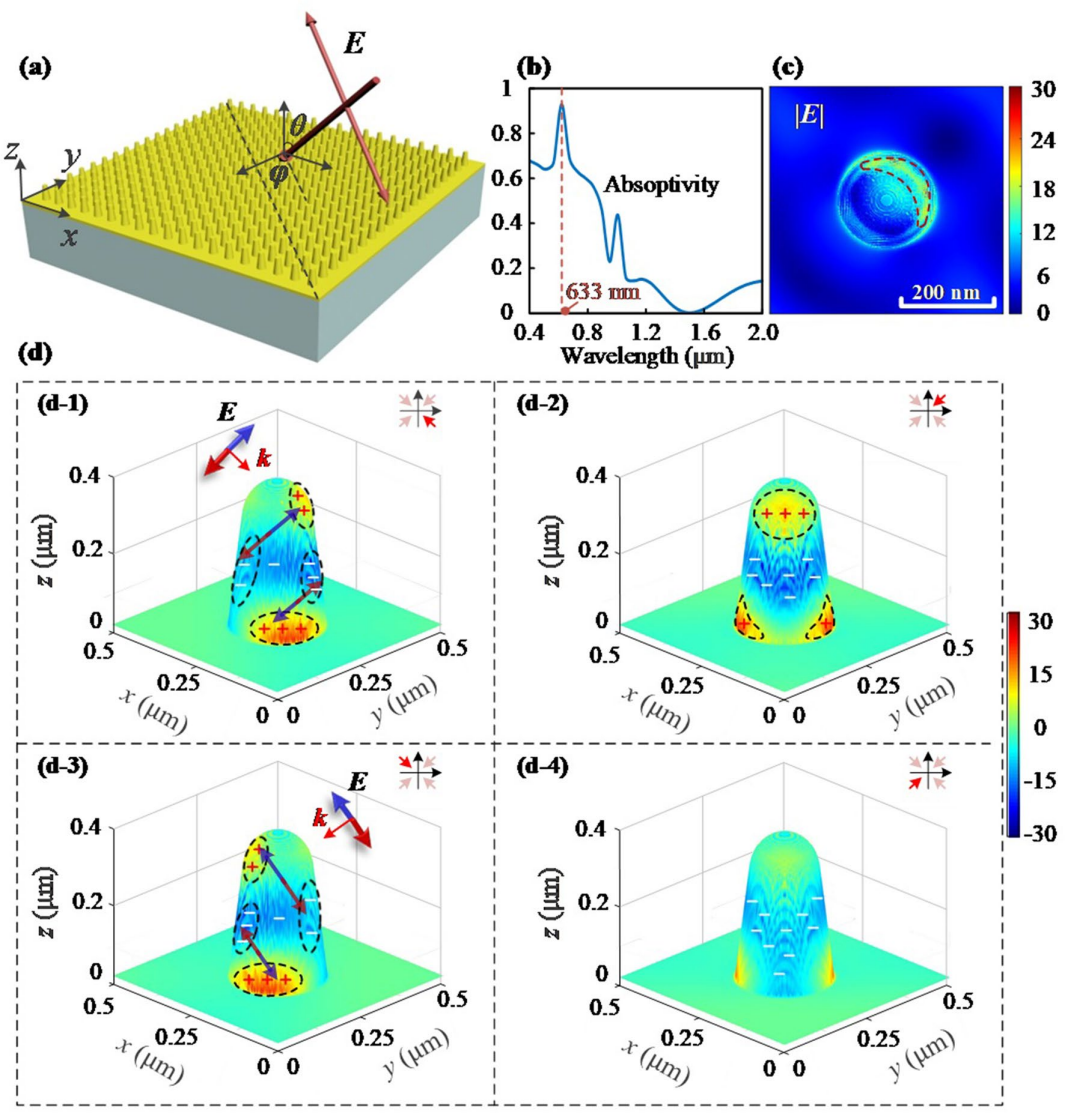
Simulated surface net charge distribution of GGNCT-III. (**a**) Schematic diagram of the simulation model with an incident angle of *θ* and the polarization angle of *φ*. (**b**) The simulated absorption spectrum in a wavelength range of 0.4–2 μm. (**c**) The top view about the distribution of the electric field intensity |***E***|. (**d**) Several typical 3D presentations about the surface net charge distribution according to different perspectives at − 45°, 45°, 135°, and − 135°, are exhibited in (**d-1**)–(**d-4**). Several small regions colored red and blue are filled by the net positive and negative charges, respectively. A detailed arrangement of a dipole of net charges over the sidewall of the GGNCT excited by the incident polarized light vector ***E*** labeled by an arrow.

To verify the theoretical prediction proposed that the SP on the surface of the nano-cone is transmitted along the wave vector, the net charge distribution on the nano-cone-tip surface under typical incident light wave excitation needs to be explored with the help of near-field experiments. Considering the limitation that the experimental device can only be incident from the top, the theoretical prediction is verified by simulation and experiment in the top-incidence mode. The GGNCT-III is selected for evaluating the net charge arrangement behaviors over the sidewall of a single GGNCT excited by the specific polarized lightwaves. A scattering near-field optical microscope (SNOM, Neaspec GmbH Co.) is used to observe the near-field behaviors of the sample, where the incident angle *θ* and the polarization angle *φ* of the laser beams of the device are both 45°, as shown in Fig. 4a. The simulations are performed according to the experimental environment. Firstly, the absorption spectrum is simulated in a broad-spectral wavelength range of 0.4–2 μm. The broadband light beams are incident over the periodic nanostructures at 45°, and thus the broadband fixed angle source technique (BFAST) is selected as a plane wave type. The PML boundary condition is selected at the *z*-axis, and the simulation period set as 0.5 μm, and the polarization angle *φ* 45°. Considering the memory size of the simulation files and the reliability of the nanostructural model, the mesh division accuracy is 2 nm, 2 nm, and 2 nm, in the *x*-, *y*-, and *z*-axes, respectively. The frequency-domain field and the power monitors are used to simulate both reflectivity and transmissivity. The reflection (*R*) and transmission (*T*) components can be obtained by direct measurement, and the absorption part is easily converted according to *A* = 1-*R*-*T* mentioned above.

From the simulation results shown in Fig. 4b, it can be seen that there is an obvious absorption peak near 633 nm. As known, the near-field light convergence enhancement at the absorption peak is the most significant, so the electric field distribution of the nanostructure mentioned at 633 nm is simulated. The difference according to the broad-spectrum simulations is that the incident light type is set at Bloch mode and the Bloch boundary conditions used in the *x*- and *y*-axes, respectively. The frequency-domain field profile monitor in 3D mode is set for obtaining the electric field distribution of the nano-cone-tip, and thus the simulation results are shown in Fig. 4c,d. Figure 4c shows the top view of the distribution of the electric field intensity |***E***| with a colorbar for presenting the electric field enhancement factor (|***E***|/|***E***_0_|) up to 30. The blue part of the figure shows the background area where the electric field component is weaker with a field enhancement factor of ~ 0.1. And the area with a stronger electric field colored by yellow and also marked by a red dashed line is distributed on the edge of one side of the nanostructure, indicating a near-field lightwave converging operation. The electric field intensity on the nanostructure is hundreds of times stronger than that in the background. Figure 4d shows the distribution of the electric field vector ***E*** on the surface of the nano-cone-tip, with (d-1)–(d-4) presenting four views at different angles of − 45°, 45°, 135°, and − 135°, which are indicated by a red arrow inserted in the upper right corner. The region with a positive electric field distribution generally means a positive net charge aggregation, and similarly the negative electric field distribution with respect to negative net charges, which are already marked by symbols “ + ” and “-” in the figure. It is shown that the positive net charges are mainly distributed at the top and bottom of one side of the nano-cone-tip, while the negative net charges are mainly distributed in the middle part and the other side. The above distribution behaviors can be attributed to the excitation of the incident electric field component, and the positive and negative net charges can also be viewed as being polarized according to the polarization direction of the incident electric field. As shown by the arrows in Figs. 4(d-1) and (d-3), two pairs of dipoles are formed on the nano-cone-tip along the traveling direction of the incident lightwaves, and also in the direction of the wave vector ***k***. This is caused by the featured transmission behaviors of lightwaves. Since the height of the nano-cone-tip is ~ 300 nm, which is almost half of the incident wavelength of 633 nm, the two pairs of dipoles are resonantly coupled so as to produce obvious absorption peaks. In summary, it can be seen that the net charges on the surface of the nano-cone-tip will make a type of dipole oscillation along the polarization direction of the incident electric field component, and then transmit along the wave vector direction, which is also a kind of energy transfer process.

**Figure 5:**
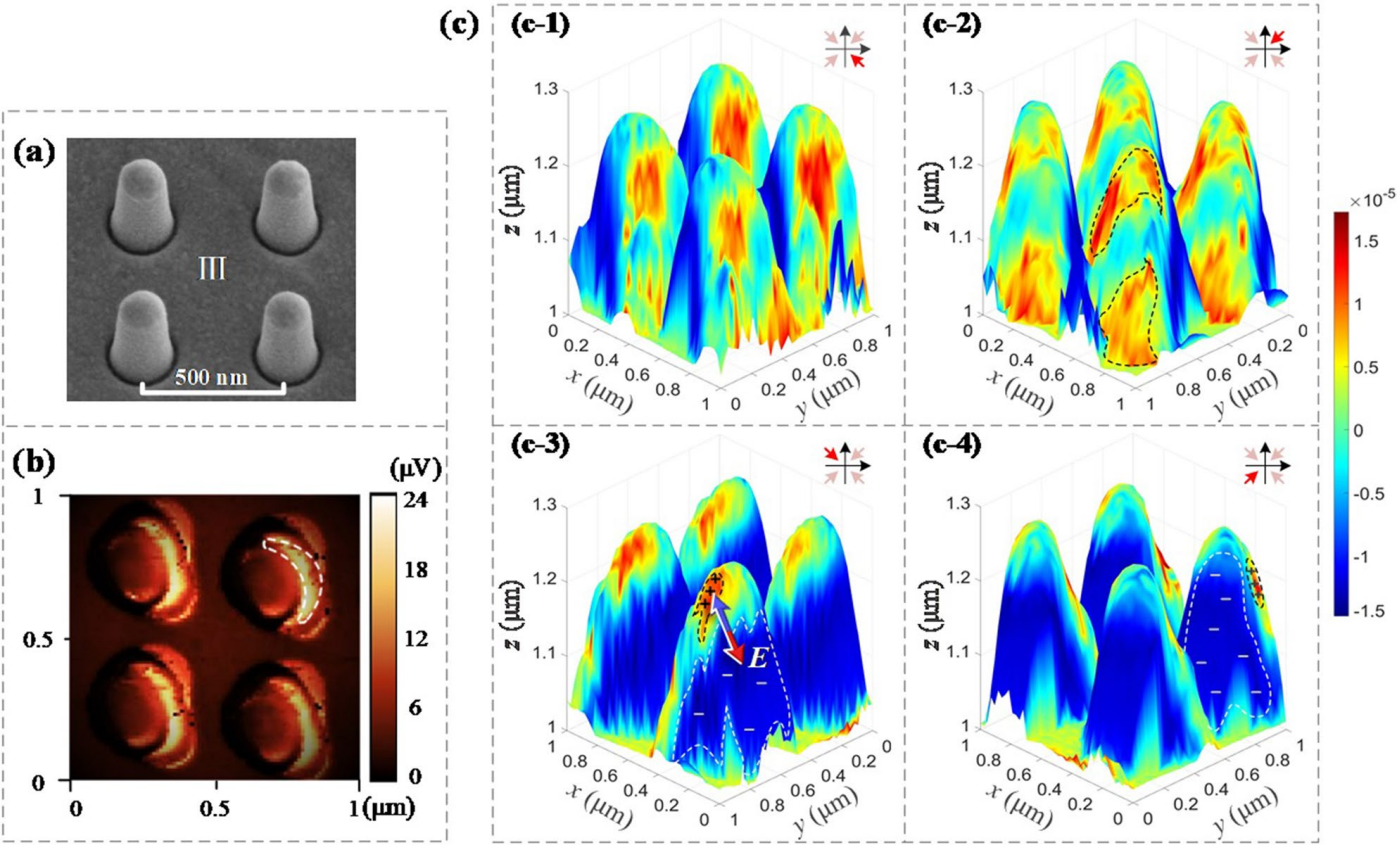
Typical surface net charge distribution of the GGNCT-III fabricated. (**a**) The SEM morphology of the GGNCT-III arrays. (**b**) The near-field light signal scanned by the probe, and the light color represents the strong signals and the dark color the weak signals. (**c**) Several typical 3D presentations about the surface net charge distribution according to different perspectives at − 45°, 45°, 135°, and − 135°, are exhibited in (**c-1**)–(**c-4**). Several small regions colored red and blue are filled by the net positive and negative charges, respectively. A detailed arrangement of a dipole of net charges over the sidewall of each GGNCT excited by the incident polarized light vector ***E*** labeled by an arrow is also indicated in (**c-3**).

As shown in Fig. 5, GGNCT-III is selected for evaluating the net charge arrangement behaviors over the sidewall of a single GGNCT excited by incident lightwaves with a needed wavelength. The SEM morphology of the GGNCT-III arrays is shown in Fig. 5a. The near-field characteristics of the sample are measured using a scattering near-field optical microscope (SNOM, Neaspec GmbH Co.). In experiments, a laser beam with a central wavelength of 633 nm is incident obliquely upon the patterned surface of the sample. Both the incidence angle *θ* and the polarization angle *φ* of the laser beams are 45°. An atomic force microscope (AFM) probe (platinum needle) is performed to obtain the near-field light vector distribution of the sample according to the initial incident direction and the polarization state of laser beams. A noncontact probe is also performed to detect the electrical signals of the near-field lightwaves excited, as shown in Fig. 5b. The light color represents the strong signals and the dark color the weak signals. The strongest signal is distributed on one side of the nano-cone-tip, which is already circled by a white dashed line and reaches ~ 24 μV, while the background signal is very weak and then closes to ~ 0.1 μV. The near-field signal on the nanostructure is hundreds of times stronger than that in the background. So, the trend of near-field distribution according to the experiments is consistent with the simulation in Fig. 4c.

The measurements are quantified in terms of the AFM topography of the nano-cone-tip, as well as the magnitude and the phase of the near-field lightwaves. Both the transient magnitude and the phase of the near-field lightwave according to SNOM measurement contain the information from the first-order to the fourth-order. Specifically, the amplitude *s*_4_ and phase *φ*_4_ of the near-field lightwaves can be extracted as effective information for evaluating the GGNCT-III. Generally, the measured near-field signals express a direct response of the surface lightwaves excited, which can be described by the following fluctuation formula of^33^:

1 $$f(x,y,t) = {\text{Re}} [s_{4} (x,y)e^{{i\varphi_{4} (x,y) - i2\pi t/T}} ]$$

where *T* represents the time period of the near-field lightwaves excited. So, the near-field light vector over the outside of each nano-cone-tip of the GGNCT-III can be obtained by the near-field measurements. It should be noted that since the surface electric field intensity is proportional to the surface net charge density, the near-field light vector character also presents the distribution behavior of the surface net charges stimulated.

Several 3D presentations based on a dipole of net charge distribution fashion over the sidewall of each GGNCT according to different perspectives at − 45°, 45°, 135°, and − 135°, which are indicated by a red arrow inserted in the upper right corner, are shown in Figs. 5(c-1)–(c-4). The red region is filled by the net positive charges and the blue the net negative charges. The net positive and negative charges are distributed over the sidewall of each GGNCT, as demonstrated in Fig. 5(c-1). The net positive charges occur at not only the sidewall top of a single GGNCT but also its bottom, as shown in Fig. 5(c-2). However, the net negative charges will disperse in a relatively large region. So, the GGNCTs will present a transient surface beam converging at the top and bottom due to many surface states constructed over the surface of each GGNCT, which can also be viewed as a special quantum dot. A detailed arrangement fashion about a dipole of net charges over the outside surface of a single GGNCT excited by the incident polarized light vector E labeled by a red arrow, which already presents the polarization orientation, is also indicated in Fig. 5(c-3). Two adjacent areas circled by two dashed lines are filled by the net positive and negative charges. As shown, the net positive charges present a relatively dense distribution with a higher charge density. However, the net negative charges or the surface "free electrons" are relatively dispersed to present a relatively low distribution density. Especially, the net charges will shape a pair of dipoles along with the polarization orientation of the incident lightwaves and oscillate according to the light vector frequency. The view shown in Fig. 5(c-4) presents the typical negative net charges distributed mainly and the dipole distribution can also be seen clearly. Unlike the simulations, only one pair of dipoles appears in the experiments. This is due to the fact that the curvature radius of the probe tip is not small enough, so as to restrict the downward movement of the probe. Therefore, the actually measured height of the nano-cone-tip is smaller than the real value of 300 nm, which is not enough to arrange the two pairs of dipoles.

**Figure 6:**
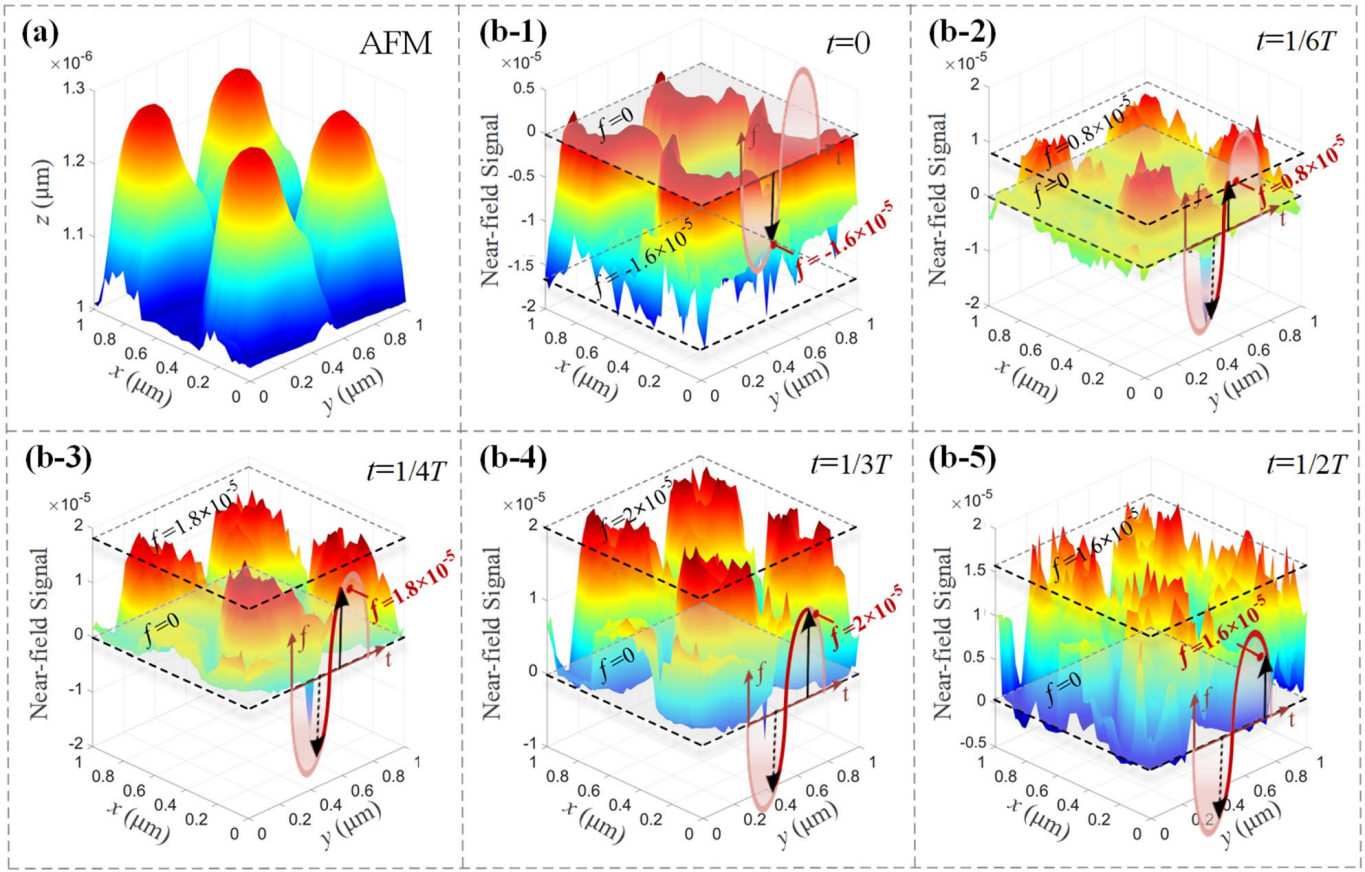
Time evolution of the near-field lightwaves excited over the outside surface of the GGNCT-III. (**a**) Surface morphology of a partial GGNCT-III. measured using AFM. (**b**) Several typical near-field lightwaves excited at different measurement moment including *t* = 0 to (**b-1**), *t* = 1/6* T* to (**b-2**), *t* = 1/4* T* to (**b-3**), *t* = 1/3* T* to (**b-4**), and *t* = 1/2* T* to (**b-5**).

To carefully analyze the dipole oscillation behaviors of the net charges generated, the time evolution characteristics of the near-field lightwaves excited over the sample of the GGNCT-III are measured in chronological order, as shown in Fig. 6. A 3D viewing of partial GGNCTs is acquired by AFM, as shown in Fig. 6a. The nano-cone-tips are colored dark blue from their bottom to Cambridge blue to light green to yellow to red for their top. The average height of the GGNCTs with a spatial period of 500 nm is ~ 250 nm. The variance behaviors of the near-field lightwaves excited in a half-time period are already plotted based on the SNOM measurement data at five specific time points: 0* T*, 1/6* T*, 1/4* T*, 1/3* T*, and 1/2* T*, respectively, as shown in Fig. 6b. The dashed plane corresponding to level 0 of the near-field measurement signal represents the reference plane corresponding to the near-field light vector *f* = 0, and also the scale plane the current near-field signal magnitude. So, a transient value on the sine curve indicated by a black arrow can be utilized to present the variation trend of the near-field lightwave function. The orientation and the magnitude of the solid black arrow mark the transient situation of the oscillation light vector, and the actual value of *f* at each moment above is − 1.6 × 10^–5^, 0.8 × 10^–5^, 1.8 × 10^–5^, 2 × 10^–5^, and 1.6 × 10^–5^. The dark red segment of the sine curve exhibits the oscillation trajectory from 0* T* to the current moment. Since the intensity of the near-field lightwaves is proportional to the surface net charge density, the variation of the near-field radiation also means the corresponding oscillation of the surface net charges. This verifies the hypothesis that the surface net charges of the nano-cone-tip will oscillate in a dipole manner.

It can be expected that when the incident lightwaves are incident vertically from the bottom of the nano-cone-tip, the net charges will make a type of dipole oscillation along the polarization direction of the incident electric field component, and then transfer to the top along the wave vector orientation. Based on a resonant response to the incident lightwaves, the light energy will continuously accumulate and finally lead to a substantial light field enhancement over the nano-cone-tips. It can be predicted that when the scale of the nanostructure along the wave vector direction is large enough, the continuously excited dipole oscillation will perform a more significant light converging enhancement at the apex.

### Signal detection by the antenna-coupled detector

**Figure 7:**
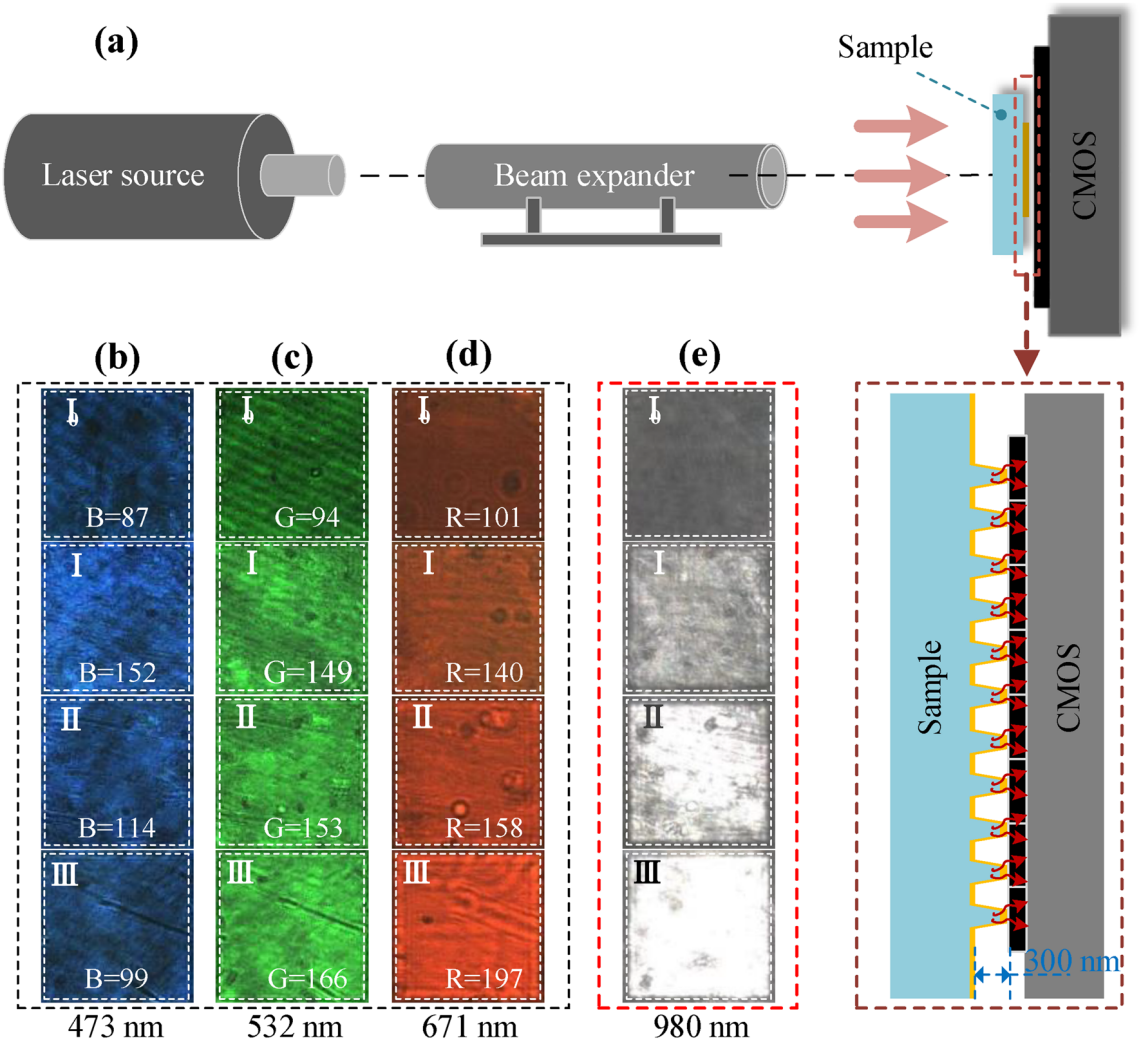
Detection of incident lightwaves at different wavelengths by CMOS sensors already coupled with three types of GGNCTs developed. (**a**) The coupling schematic diagram of the GGNCTs and an arrayed CMOS sensor. (**b**) to (**e**) The typical images detected at different light wavelengths including 473 nm, 532 nm, 671 nm, and 980 nm. The labels of I _0_ and I to III indicate the images detected by the detector coupled to the different functional structures including the unstructured sample, the functional GGNCT-I, the GGNCT-II, and the GGNCT-III. The graphic brightness represents the strength of the imaging signal. To quantify the brightness, the pixel mean values of the R, G, and B channels are taken for the red, green and blue images respectively, and the corresponding values are inserted in each image.

The constructed GGNCT presents a strong localized effect of the ***E***-field at the top, resulting in an evident increase of the excited near-field lightwave intensity by 4 orders of magnitude over incident lightwaves. Therefore, an effective detection method is proposed for performing a near-field coupling between the nano-antenna and the photosensitive surface of the complementary metal–oxide–semiconductor (CMOS) sensors for detecting weak lightwaves in a relatively broad wavelength range. The experiments initially validate the idea above. A semifinished product of a back-illuminated CMOS chip (Model: C2395, HYLIX) with a 3 μm × 3 μm sensitive size and 2 megapixels, whose photosensitive surface is exposed directly, is utilized for performing weak light detection experiments. The sample surface with nano-cone-tips and the photosensitive surface of the CMOS sensor are laminated together using nanospheres as spacers. The sample is matched onto the CMOS sensor to directly detect the imaging signals through the GGNCTs as an arrayed optical antenna to efficiently receive and converge incident lightwaves at different wavelengths, as shown in Fig. 7. The distance between the sample and the CMOS sensors can be adjusted according to the size of the nanospheres. Here we use nanospheres with a diameter of 300 nm. According to the design, the height of the nano-cone-tip is ~ 300 nm as well, so as to ensure the nano-cone-tip almost touches the photosensitive surface, and thus achieve a needed near-field coupling. The sensor is soldered onto the mobile industry processor interface MIPI (UM330, DOTHINKEY), which is directly connected to the PC via USB 3.0 interface and the detection signal is visualized by special software. The coupling schematic diagram of the GGNCTs and the CMOS sensors is shown in Fig. 7a, and an enlarged viewing is inserted. A laser beam is directly incident on the sample and then detected by an arrayed CMOS sensor, which is closely attached to the sample.

A typical absorption peak such as at the wavelength of 980 nm of the GGNCT-III, is selected for imaging detection experiments. In addition, considering the case that the red–green–blue (R-G-B) spectroscopic system is usually used for imaging evaluation, the typical central wavelengths of 671 nm, 532 nm, and 473 nm, are also selected. Figure 7b–e show the actual images measured at different wavelengths of 473 nm, 532 nm, 671 nm, and 980 nm. The labels I _0_ and I to III indicate the images detected by the detector coupled to the different functional structures including the unstructured sample, the functional GGNCT-I, GGNCT-II, and GGNCT-III. The three kinds of GGNCTs all have a spatial period of 500 nm and size of 1000 × 1000 pixels. The graphic brightness presents the light intensity detected. To quantify the brightness, the pixel mean values of the R, G, and B channels are taken for the red, green and blue images respectively, and the corresponding values are inserted in each image. The larger the mean value, the stronger the detection signal of the corresponding color. As demonstrated, when detecting blue light at 473 nm, the pixel mean values of the B channels in regions I _0_ to III are 87, 152, 114 and 99. The detector coupled to GGNCT-I detects the most vigorous light intensity, then followed by GGNCT-II, and the smallest is GGNCT-III. To 532 nm light beams, the pixel mean values of the G channels in regions I _0_ to III are 94, 149, 153 and 166. There exists a slight difference between the three detected images, and the light intensity of the -III is slightly stronger. To the red beams with a central wavelength of 671 nm, the pixel mean values of the R channels in regions I_0_ to III are 101, 140, 158 and 197. There is an enhanced trend of the regional brightness according to a sequence of the -I to -II to -III. And the same trend is taken at the infrared lightwaves with a central wavelength of 980 nm, even more obviously than 671 nm wavelength. It is worth pointing out that for all measurement wavelengths, the intensity of the detection signal in the coupled structure region is significantly higher than that in the unstructured region.

**Figure 8:**
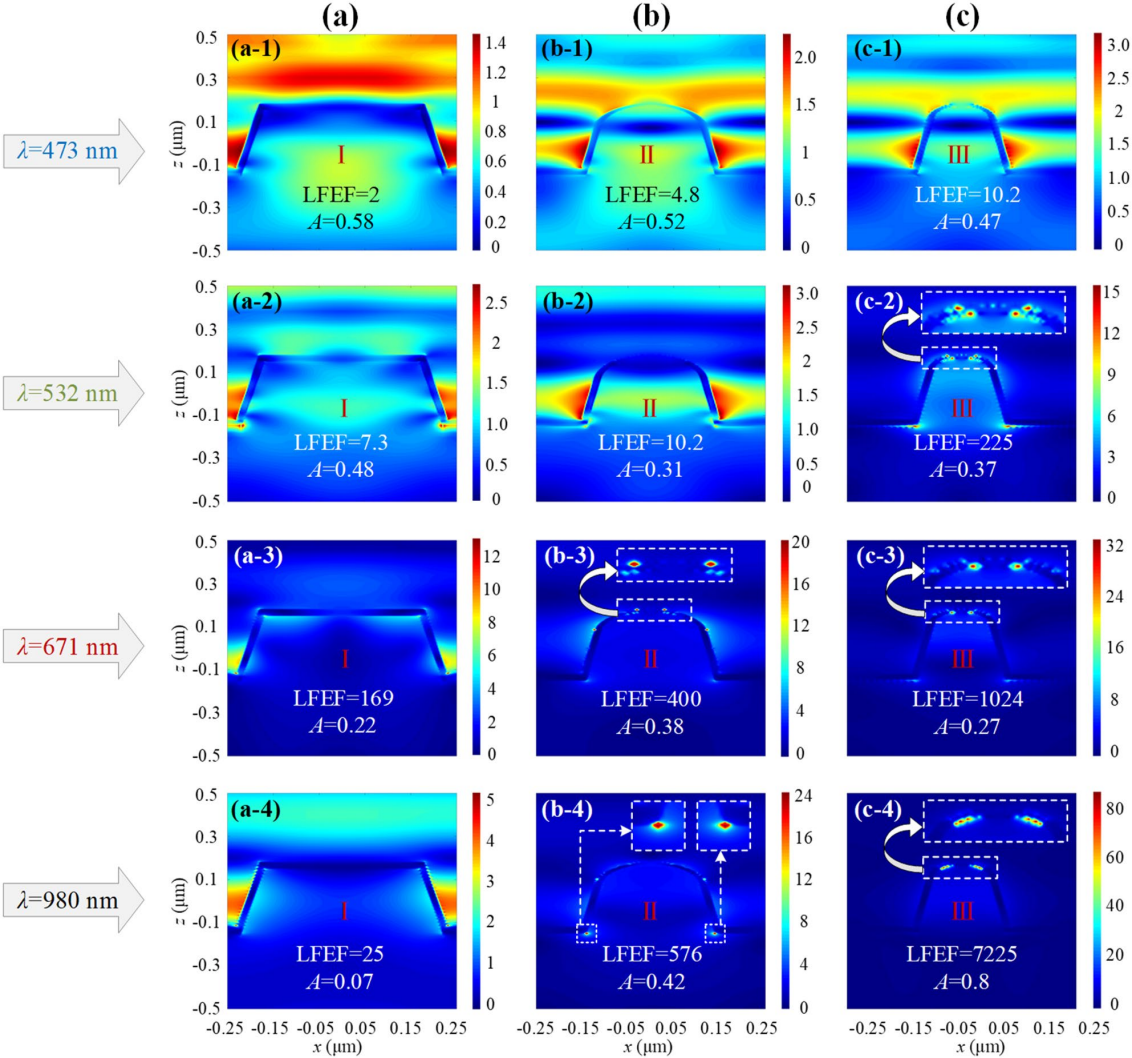
The ***E***-field distributions of the GGNCTs at several typical wavelengths selected, such as 473 nm, 532 nm, 671 nm and 980 nm. (**a**) The ***E***-field profiles of GGNCT-I at 473 nm, 532 nm, 671 nm and 980 nm as shown in (**a-1**)–(**a-4**). The values of *LFEF* at several typical wavelengths are 2, 7.3, 169, and 25, and the values of absorptivity *A* are 0.58, 0.48, 0.22, and 0.07, respectively. (**b**) The ***E***-field profiles of GGNCT-II at 473 nm, 532 nm, 671 nm and 980 nm as shown in (**b-1**)–(**b-4**). The values of LFEF at several typical wavelengths are 4.8, 10.2, 400, and 576, and the values of absorptivity *A* are 0.52, 0.31, 0.38, and 0.42, respectively. (**c**) The ***E***-field profiles of GGNCT-III at 473 nm, 532 nm, 671 nm and 980 nm as shown in (**c-1**)–(**c-4**). The values of LFEF at several typical wavelengths are10.2, 225, 1024, and 7225, and the values of absorptivity *A* are 0.47, 0.37, 0.27, and 0.8, respectively. The enlarged views of the strong near-field convergence spot are inserted in the corresponding figures.

To further analyze the intrinsic reasons for the intensity difference of the images obtained experimentally, the electric field distributions of the three nanostructures at the corresponding wavelengths are simulated, as shown in Fig. 8. The values of the corresponding local light field enhancement factor LFEF and absorptivity *A* are given at each wavelength to help analyze the near-field convergence effects of the three nanotips GGNCTs- I, -II, and -III. Figures 8(a-1)–(a-4) show the ***E***-field profiles of GGNCT-I at typical wavelengths of 473 nm, 532 nm, 671 nm, and 980 nm. The values of LFEF at several typical wavelengths are 2, 7.3, 169, and 25, and the values of absorptivity *A* are 0.58, 0.48, 0.22, and 0.07, respectively. It can be seen that there is no strong ***E***-field convergence effect at the top of GGNCT-III at any of the four typical wavelengths. Figures 8(b-1)–(b-4) show the ***E***-field profiles of GGNCT-II at typical wavelengths of 473 nm, 532 nm, 671 nm, and 980 nm. The values of LFEF at several typical wavelengths are 4.8, 10.2, 400, and 576, and the values of absorptivity *A* are 0.52, 0.31, 0.38, and 0.42, respectively. It can be seen that the convergence effect appears at the top and bottom of GGNCT-II at typical wavelengths of 671 nm and 980 nm, where the LFEF is relatively large. Figures 8(c-1)–(c-4) show the ***E***-field profiles of GGNCT-III at typical wavelengths of 473 nm, 532 nm, 671 nm, and 980 nm. The values of LFEF at several typical wavelengths are10.2, 225, 1024, and 7225, and the values of absorptivity *A* are 0.47, 0.37, 0.27, and 0.8, respectively. It can be seen that at typical wavelengths of 532 nm and 671 nm, the convergence effect appears at the top and bottom of GGNCT-III, where the LFEF are relatively large. While at 980 nm, the ***E***-field all converges at the top of GGNCT-III forming a pair of very strong dipoles, and the LFEF reaches a high value of 7225. In summary, the near-field convergence effect is gradually significant with the increase of LFEF, and converges at the top when LFEF and *A* reach the maximum at the same time.

At the wavelength of 473 nm, the LFEF of the three kinds of GGNCTs is at a very low value. However, the absorptivity of GGNCT-I is the highest, followed by GGNCT-II and GGNCT-III. High absorptivity will lead to a relatively strong local field on the sidewall of the nano-cone-tip, which is the reason why the detector coupled to GGNCT-I detects the strongest signal. At the wavelength of 532 nm, the LFEF of GGNCT-III is relatively larger than that of GGNCT-I and GGNCT-II, and there is a relatively large near-field convergence effect at the top and bottom of the cone, which leads to the strongest detection signal. At the wavelength of 671 nm and 980 nm, the value of LFEF increases according to a sequence of the -I to -II to -III. GGNCT-III obtains high values of 1024 and 7225 respectively, and the field enhancement effect is significant. Especially at 980 nm, the LFEF and the absorptivity *A* of GGNCT-III reach the maximum at the same time, and the ***E***-field is strongly localized at the top of the nano-cone-tip. The strong local near-field will be directly received by the photosensitive element of the CMOS, forming a relatively strong detection signal. Considering the case with greater sharpness and the smaller transverse dimension of a single nano-cone-tip in the GGNCT-III, the net charges can be effectively squeezed towards the apex through SP propagation along the sidewall and thus leading to a denser net charge distribution, which means a larger surface light field enhancement. It can be speculated that by properly configuring the structural size of the nano-cone-tips, an obvious lightwave nano-converging performance can be obtained at the desired wavelength, so as to effectively achieve a localized amplification of very weak incident lightwaves. It lays a foundation for subsequent research on infrared imaging detection.

## Conclusions

In summary, the GGNCTs developed as an arrayed optical antenna can excite SPs in a dipole form and converge towards the apex. The LFEF can be enhanced to a high value of ~ 2 × 10^4^, and the most substantial light absorption peak of ~ 98% is obtained at the same wavelength. The near-field measurements at the wavelength of 633 nm show that the surface net charges distributed over a single GGNCT make a typical dipole of resonant oscillation and the energy transmission along the wave vector orientation, thus leading to a strong local light field enhancement around the tip. The effective detection method we proposed by performing a near-field coupling between the nano-antenna array and CMOS sensors can achieve weak lightwave detection in a wide spectral range. In this case, the nano-antenna array can be considered as a special optical filter placed in front of the CMOS pixels, facilitating the CMOS pixels to be more capable of detecting the enhanced weak target signals. The experiments based on coupling CMOS sensors with the GGNCT array to detect different lightwaves at wavelengths such as 473 nm, 532 nm, 671 nm, and 980 nm, show a remarkable character that a better capability about the net charge adjusting and aggregating can be obtained at the absorption peak of the GGNCTs, and also the stronger detected signals can be acquired. It can be predicted that by properly configuring the structural size of the nano-cone-tips, a significant lightwave nano-converging performance can be realized at the desired wavelength for achieving a very weak light signal amplification and detection for an imaging application. It highlights the continuous development of infrared photosensitive and imaging methods and technology.

## Methods

### Sample fabrication

The glass material is selected for the formation of the glass-based GGNCT array mainly by EBL and ICP etching.

At first, a photoresist array was fabricated over the glass wafer via electron beam lithography (EBL: JBX 6300FS, JEOL). During the EBL exposure, the exposure pattern was set to be nano-blocks (300 nm × 300 nm for GGNCT-I, 200 nm × 200 nm for GGNCT-II, 150 nm × 150 nm for GGNCT-III,) with an arranged frequency of 500 nm. To ensure EBL exposure accuracy, 5 nm aluminum was sprayed on the glass substrate as a conductive layer at first. Then the spin processor was conducted. The parameters were set to 40 s and 4000 rpm to form a layer of tackifier (AR 300-80) with a thickness of 20 nm for enhancing the adhesion between the wafer and the photoresist. After spin-coating, the sample was baked at 180 °C for 2 min. Next, the negative photoresist (ma-N2403) with a thickness of 380 nm was formed with the spin parameters of 40 s and 2000 rpm, and afterward baked at 90 °C for 1 min. Furthermore, the arrayed photoresist mask was defined via EBL exposure and then developed by Tetramethylammonium Hydroxide (TMAH) for 2 min to acquire the needed pattern on the silicon substrate.

Secondly, the inductively coupled plasma etching (ICP: Oxford Plasma Pro System 100 ICP 380) was adopted to fabricate the nano-cone-tips array upon the wafer. The reaction gas and concentration parameters used were O_2_ for 5 sccm and C_4_F_8_ for 45 sccm. The process parameters, including the pressure, the ICP power, the RF power, the reaction temperature, and the etching time were set to 3.7 mTorr, 1200 W, 50 W, 20 °C and 80 s, respectively. Then, the sample was heated in a water bath of NMP solution at 80 °C for 30 min to remove the unetched photoresist. After degumming, the reactive ion etching (ME-3A) is conducted to shape a relatively sharp sidewall.

Finally, the magnetron sputtering (Kurt J. Lesker LAB 18) is utilized to deposit a 30 nm thick gold film over the patterned surface of the glass substrate with a 5 nm thick titanium as adhesive.

### Near-field measurements

The experimental setup of SNOM measurement is briefly described below. The *p*-polarized incident beams with a central wavelength of ~ 633 nm were incident obliquely along the diagonal with an incident angle of 45° upon a horizontal facet, the same as that in Fig. 4a. During the measurement, the incident beams were focused via a parabolic mirror onto both the sample and the platinum AFM probe oscillating vertically, which was also applied as a scattering source and its tip-scattered light was modified by the near-field lightwave properties of the sample below the tip. Then, the tip-scattered light was demodulated at the *n-*th harmonics of the tapping frequency yielding background-free images. To filter out the background signal, *n* = 3 was chosen in this work. Finally, both the amplitude and phase of the tip-scattered light, delivering the information about the near-field electric-field signals, were recovered via all-optical interferometric detection.

### Absorption spectrum measurements

The dispersive spectrometer produced in HORIBA (model iHR550), has the following parameters: focal length of 550 mm, resolution of 0.06 nm, and grating size of 76 mm × 76 mm. The spectral range of the spectrometer is from 0.4 to 2.0 μm, which already covers the visible and partial near-infrared bands. The reflection (*R*) and transmission (*T*) components can be obtained by direct measurements, and the absorption part converted according to the relation of *A* = 1 − *R* − *T*.

### Signal detection

A CMOS bare chip (model: C2395, HYLIX) with a 3 μm × 3 μm sensitive size and 2 megapixels, is soldered onto the mobile industry processor interface MIPI (UM330, DOTHINKEY), which is directly connected to the PC via USB 3.0 interface and the detection signal visualized by special software. The experiments were conducted on the built platform. Adjust the optical axes of the laser, beam expander, and antenna-coupled detector to the same line. Then turn on the laser and get the detection signal picture on the PC. Then switch to a laser of a different wavelength and do the same.

## Data Availability

Data underlying the results presented in this paper are not publicly available at this time but may be obtained from the authors upon reasonable request.

## References

[1] Li H, Wang X, Wang S (2019). Realization and characterization of terahertz surface plasmon light capsules. Appl. Phys. Lett..

[2] Shang Q, Shuai Z, Jie C, Yang P, Zhang Q (2018). Surface plasmon enhanced strong exciton-photon coupling in hybrid inorganic-organic perovskite nanowires. Nano Lett..

[3] Jing D, Wang J (2017). Design and fabrication of hybrid SPP waveguides for ultra high-bandwidth low-penalty terabit-scale data transmission. Opt. Express.

[4] Wang S, Yoo S, Zhao S (2021). Gate-tunable plasmons in mixed-dimensional van der Waals heterostructures. Nat. Commun..

[5] Ding F, Yang Y, Deshpande RA, Bozhevolnyi SI (2018). A review of gap-surface plasmon metasurfaces: Fundamentals and applications. Nanophotonics.

[6] Wei H, Pan D, Zhang S (2018). Plasmon waveguiding in nanowires. Chem. Rev..

[7] Nagy BJ, Pápa Z, Péter L (2020). Near-field-induced femtosecond breakdown of plasmonic nanoparticles. Plasmonics.

[8] Song B, Ganjeh Y, Sadat S (2015). Enhancement of near-field radiative heat transfer using polar dielectric thin films. Nat. Nanotech..

[9] Karaagac H, Islam MS (2014). Enhanced field ionization enabled by metal induced surface states on semiconductor nanotips. Adv. Funct. Mater..

[10] Neubrech F, Huck C, Weber K, Pucci A, Giessen H (2017). Surface-enhanced infrared spectroscopy using resonant nanoantennas. Chem. Rev..

[11] Ahn B (2016). Optimization of a nanotip on a surface for the ultrafast probing of propagating surface plasmons. Opt. Express.

[12] Yang J, Sun Q, Ueno K (2018). Manipulation of the dephasing time by strong coupling between localized and propagating surface plasmon modes. Nat. Commun..

[13] Park C, Oh S, Hahn JW (2019). Theoretical analysis of high-efficient dielectric nanofocusing for the generation of a brightness light source. Sci. Rep..

[14] Jensen RA (2016). Optical trapping and two-photon excitation of colloidal quantum dots using bowtie apertures. ACS. Photon..

[15] Schnell M (2009). Controlling the near-field oscillations of loaded plasmonic nanoantennas. Nat. Photon..

[16] Liang Y, Koshelev K, Zhang F (2020). Bound states in the continuum in anisotropic plasmonic metasurfaces. Nano. Lett..

[17] Liang Y, Lin H, Koshelev K (2021). Full-stokes polarization perfect absorption with diatomic metasurfaces. Nano Lett..

[18] You E (2021). Nanobridged rhombic antennas supporting both dipolar and high-order plasmonic modes with spatially superimposed hotspots in the mid-infrared. Opto-Electron. Adv..

[19] Deeb C, Guo Z, Yang A, Huang L, Odom TW (2018). Correlating nanoscopic energy transfer and far-field emission to unravel lasing dynamics in plasmonic nanocavity arrays. Nano Lett..

[20] Gross H, Hamm JM, Tufarelli T, Hess O, Hecht B (2018). Near-field strong coupling of single quantum dots. Sci. Adv..

[21] Salfi J (2018). Valley filtering in spatial maps of coupling between silicon donors and quantum dots. Phys. Rev. X.

[22] Guo J, Jian J, Wang D, Zhang X (2020). Controlling amplified spontaneous emission of quantum dots by polymerized nanostructure interfaces. Opt. Let..

[23] Duchet M (2021). Femtosecond laser induced resonant tunneling in an individual quantum dot attached to a nanotip. ACS Photonics.

[24] Etman N, Said AMA, Atia KSR (2019). Quantum effects in imaging nano-structures using photon-induced near-field electron microscopy. Sci. Rep..

[25] Guo Y, Zhang Z, Pu M (2019). Spoof plasmonic metasurfaces with catenary dispersion for two-dimensional wide-angle focusing and imaging. Iscience.

[26] Yokogawa S, Burgos S, Atwater H (2012). Plasmonic color filters for CMOS image sensor applications. Nano. Lett..

[27] Park KD (2016). Hybrid tip-enhanced nano-spectroscopy and -imaging of monolayer WSe_2_ with local strain control. Nano. Lett..

[28] Park KD, Raschke MB (2018). Polarization control with plasmonic antenna-tips: A universal approach for optical nano-crystallography and vector-field imaging. Nano Lett..

[29] Li P, Lewin M, Kretinin A (2015). Hyperbolic phonon-polaritons in boron nitride for near-field optical imaging and focusing. Nat. Commun..

[30] Hu F, Luan Y, Zhe F, Palubski I, Ba Sov DN (2017). Imaging the localized plasmon resonance modes in graphene nanoribbons. Nano Lett..

[31] Zheng D, Wang J, Hu W (2016). When nanowires meet ultrahigh ferroelectric field-high-performance full-depleted nanowire photodetectors. Nano Lett..

[32] Zhang J, Guo Z, Ge C (2015). Plasmonic focusing lens based on single-turn nano-pinholes array. Opt. Express.

[33] Schnell M, Garcia-Etxarri A, Huber AJ (2009). Controlling the near-field oscillations of loaded plasmonic nano-antennas. Nat. Photon..

